# MicroRNA-96-5p represses breast cancer proliferation and invasion through Wnt/β-catenin signaling via targeting CTNND1

**DOI:** 10.1038/s41598-019-56571-z

**Published:** 2020-01-08

**Authors:** Xiao-hui Gao, Ya-li Zhang, Zhi-ye Zhang, Shuang-shuang Guo, Xiao-bing Chen, Yan-zhen Guo

**Affiliations:** 1 Department of Oncology, The First Affiliated Hospital of Henan University of Science and Technology, Henan province, 471000 China; 2 Department of Oncology, Henan Cancer Hospital, Henan province, 471000 China

**Keywords:** Breast cancer, Prognostic markers

## Abstract

Low miR-96-5p expression is characteristic of many cancers but its role in breast cancer (BCa) remains poorly defined. Here, the role of miR-96-5p in BC development was assessed. We demonstrate that exogenously expressing miR-96-5p inhibits the proliferative, migratory and invasive capacity of BCa cells. Mechanistically, miR-96-5p in BCa cells was found to target and downregulate catenin delta 1 (CTNND1) leading to decreased β-catenin expression, a loss of WNT11 signaling, reduced cyclin D1 levels and lower MMP7 expression. Exogenously expressing CTNND1 alleviated these effects. In summary, we are the first to reveal that miR-96-5p inhibits the proliferative, invasive and migratory phenotypes of BCa cells the targeting of CTNND1 and subsequent Wnt/β-catenin signaling. These data highlight miR-96-5p as a novel target for BC treatment.

## Introduction

Of all life-threatening human cancer cases, breast cancer (BCa) remains the most commonly diagnosed^1^. In the United States (US), nearly 330,000 new cases of BC occurred in 2017 over a third of which were invasive^2^. Despite great strides to improve BCa diagnostics and therapeutics, the rates of mortality remain high with over 40000 deaths occurring in 2017 in the US alone^2^. BCa cells possess a remarkable ability to metastasize to the bone marrow, the regional lymph nodes, liver, and the lungs forming a distinct tissue microenvironment (TME) that permits efficient dissemination from the site of the primary tumor^3,4^. Identifying the molecular mechanisms controlling BCa progression can thus inform the design of more efficacious anti-BCa therapies.

It is now well-accepted that the dysregulation of cellular MicroRNAs (miRNAs) leads to a range of pathological processes in animals and humans mediated through their ability to bind protein-coding transcripts^5^. An increasing body of evidence points to the presence of a link between miRNAs to the initiation, development, and metastasis of various types of tumors, including BC^6–9^. Although miR-96-5p is implicated in multiple cancers^10–12^, little is known regarding its relationship to the development and progression of BC.

Here, we assessed the contribution of miR-96-5p to BCa and reveal its downregulation in BCa cells. We demonstrate that exogenously increasing miRNA-95-5p expression inhibits the proliferative and metastatic potential of BCa cells which is in part, mediated through the targeting of catenin delta 1 (CTNND1) and subsequent Wnt/β-catenin signaling. We thus highlight miR-96-5p as a novel therapeutic for much needed anti-BCa treatments.

## Methods

### Tissues and cells

We collected 155 BCa samples and non-tumor tissues. Included patients received no prior chemotherapy, immunotherapy, or radiotherapy prior to the surgery. Samples were verified as BCa for an experienced pathologist and frozen in liquid N2 prior to use. All patients signed an informed consent form. The protocol of the study was reviewed and approved by the Institutional Human Experiment and Ethic Committee of the First Affiliated Hospital of Henan University of Science and Technology and the entire investigation was compliant with the Helsinki Declaration.

MDA-MB-246, MDA-MB-231, T47D and ZR-75-30 cells were used as model BCa lines (Chinese Cell Bank). Cells were cultured in DMEM plus 10% FBS and 1% (v/v) pen/strep at 37 °C in a 5% CO_2_ humidified atmosphere. The normal epithelial breast cell line MCF-10A was cultured in MEBM basal medium (Lonza, Basel, Switzerland) supplemented with the MEGM Single Quot Kit (Lonza) and cholera toxin (List Biological Labs, Campbell, CA).

### Cell transfection

Negative controls and MiR-96-5p mimics were purchased from Sigma. CTNND1 expression plasmid (LV-CTNND1), small interfering RNA (siRNA) targeting CTNND1(sh-CTNND1) and non-targeting (vector) siRNA were purchased from GenePharma (Shanghai, China).Cells were transfected using Lipofectamine 3000 (Invitrogen) as per the recommended protocols (100 nM of miRNAs per sample). Transfections were confirmed by q-RT-PCR analysis and repeated on a minimum of 3 occasions.

### qRT-PCR analysis

BCa cells were lysed in Trizol for RNA isolation (Invitrogen). cDNA was generated using commercial RT kits (miScript; QIAGEN). miR-96-5p and CTNND1 mRNA were amplified utilizing Sequence Detection Model 7500 (Applied Biosystems). QRT-PCRs were performed using MiScript SYBR Green (QIAGEN) and SsoAdvanced Universal SYBR Green (Bio-Rad). Relative expression was assessed using the 2^−ΔΔCt^ method and normalized to U6/GAPDH (see Table 1 for primers used in the study).

**Table 1:** Primer sequences.

miR-96-5p	forward, 5′-ACGATGCACCTGTACGATCA-3′
	reverse, 5′-TCTTTCAACACGCAG GACAG -3′
CTNND1	forward, 5′-ATGTTTGCGAGGAAGCCGC-3′
	reverse, 5′-CGAGTGGTCCCATCATCTG-3′
GAPDH	forward, 5′-CCATGTTCGTCATGGGTGTG-3′
	reverse, 5′-GGTGCTAAGCAGTTGGTGGTG-3′
U6	forward, 5′-GCTTCGGCAGCACATATACTAAAAT-3′
	reverse, 5′-CGCTTCACGAATTTGCGTGTCAT-3′

### Western blot analysis

BCa cells/tissues were harvested in RIPA buffer and protein content was determined via BCA Assays (Bio-Rad). Lysates were resolved via SDS-PAGE electrophoresis and proteins semi-dry transferred to nitrocellulose membranes (Invitrogen). Membranes were blocked and labeled with antibodies targeting WNT11, CTNND1, CyclinD1, β-catenin and anti-MMP7 (all purchased from Abcam) and secondary HRP-conjugated antibodies (Cell Signaling Technologies). GAPDH was probed as a loading control. Proteins were visualized using ECL (Millipore).

### Cell mobility and invasiveness

In Boyden-type chambers, the upper wells were left uncoated (for migration assays) or coated (for invasion assays) with Matrigel (BD Bioscience). Chambers were seeded with 1 × 10^4^ cells and incubated in DMEM lacking serum. DMEM containing 10% FBS was also added to the chambers. After 24 h at 37 °C, cotton swabs were used to removed cells still resident in the upper chambers, whilst translocated cells were PFA fixed and hematoxylin-stained. Stained counted via microscopy.

### Cell proliferation assay

BCa cells (1 × 10^4^/mL) were treated with CCK-8 (Dojindo) for 2 h, and absorbances were read at 450 nm. Assessments were performed every 24 h over a 3-day period.

### Clone formation assay

BCa cells (~500 cells per 6-well plate) were assessed for colony formation after 14 d via 0.5% crystal violet staining.

### MiR-96-5p-binding assays

The 3′UTR of CTNND1 was amplified and cloned into the pGL3 (Promega). MiR-96-5p-binding site mutants were generated via Quick-change (New England Biolabs). Cells were transfected with PGL3-WT or mutant CTNND1 constructs with miR-96-5p inhibitors/mimics. Dual Luciferase reporter assays were performed on a luminometer (Berthold Detection System, Promega). Firefly luciferase values were normalized to Renilla controls.

### Statistical analyses

Graphpad 5.0 was employed for intra-group comparisons. Continuous variables are shown as means ± SD. QRT-PCR assays, luciferase data, clone formation and transwell assays were compared using a Student’s t-test. A two-way ANOVA was employed to evaluate BCa cell growth curves. Chi-square tests were used to assess clinical features relative to miR-96-5p levels. Kaplan Meier (KM) curves were used for survival assessments and compared via log-rank tests. Cox-regression models were used for univariate/multivariate comparisons. P-values < 0.05 were considered significant (n = 3 for all experiments).

## Results

### Low levels of miR-96-5p expression in BCa

**Figure 1:**
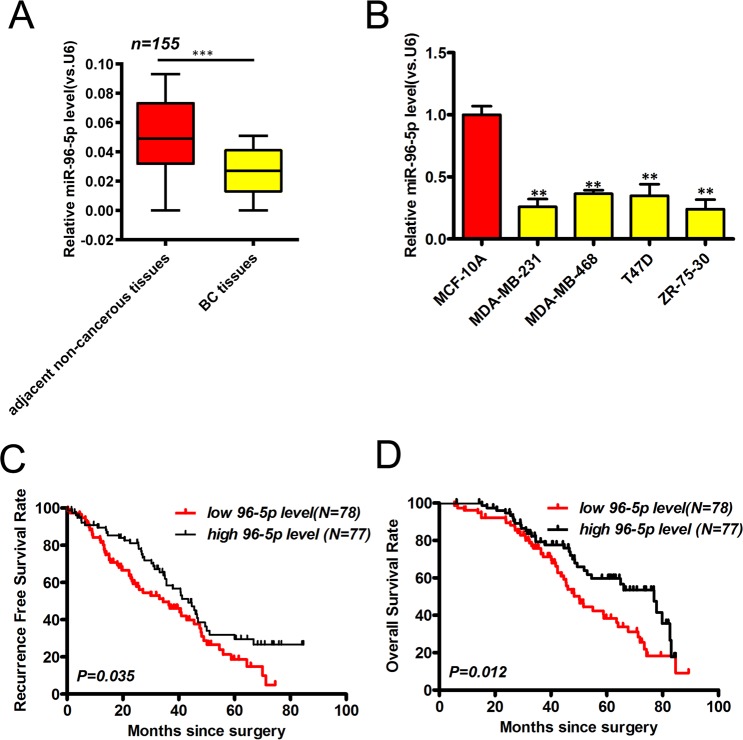
MiR-96-5p in BCa. (**A**) Expression in BCa tissue assessed by qRT-PCR (n = 155). (**B**) Expression in BCa and normal intestinal epithelium cell lines. (**C**,**D**) Patient OS (**C**) and RFS (**D**) according to miR-96-5p levels assessed by KM curves (log-rank test). N = 3; *p < 0.05, **p < 0.01, and ***p < 0.001.

MiR-96-5p expression in BCa samples *vs*. non-cancerous adjacent tissues were compared via qRT-PCR. Compared to non-cancerous tissue, miR-96-5p levels were dramatically reduced in BCa samples (P < 0.05, Fig. 1A). Consistent with this finding, low levels of miR-96-5p expression were detected in all BCa cells compared to the normal epithelial breast cell line MCF-10A cells (P < 0.05 vs. each BCa cell line, Fig. 1B). These data highlight the significant suppression of this miRNA in BCa.

### MiR-96-5p expression correlates with BCa phenotypes

To characterize BCa phenotypes according to miRNA expression, two miR-96-5p subgroups were formed (high *vs*. low groups). As shown in Table 2, a significant correlation of reduced miR-96-5p expression in BCa with distant metastasis (P = 0.003) and TNM stage (P < 0.001) were evident suggesting a direct link to BCa progression. We further examined the association of miR-96-5p with BCa survival. In the low miR-96-5p group, recurrent-free survival (RFS) was 34.40 months compared to 43.40 months in the high-expression group, which significantly differed (low group vs. high group; P = 0.036; 95% CI: 1.03 to 2.32; Fig. 1C). Similarly, significant differences in the media overall survival (OS) of low- and high-expression groups were also observed (50.20 vs 76.90 months, respectively; low group vs. high group; P = 0.012; 95% CI: 1.13 to 2.74; Fig. 1D). Cox regression analysis of the 155 patients in the study cohort showed that both TNM staging and miR-96-5p levels could predict the OS and RFS of BCa patients (Table 3). Collectively, these data inferred a causative role for the loss of miR-96-5p expression for malignant BCa.

**Table 2:** Association between miR-96-5p expression and the clinicopathologic characteristics of BCa patients in the study cohort.

Characteristics	n	miR-96-5p expression level		P Value
		Low level n = 78	High level n = 77	
**Age**				
≥50	62	30	32	0.694
<50	93	48	45	
**Menopause**				
Yes	60	29	31	0.694
No	95	49	46	
**Pathological Grade**				
I + II	133	66	67	0.669
III	22	12	10	
**Estrogen receptor**				
Positive	90	45	45	0.925
Negative	65	33	32	
**Progesterone receptor**				
Positive	86	43	43	0.929
Negative	69	35	34	
**Her-2**				
Positive	44	21	23	0.684
Negative	111	57	54	
**TNBC**				
Yes	17	9	8	0.819
No	138	69	69	
**Distant metastasis**				
No	137	63	74	**0.003**
Yes	18	15	3	
**TNM stage**				
I + II	105	41	64	**<0.001**
III	50	37	13	

Pearson chi-square tests were used to compare subgroups. Bold values are less than 0.05.

**Table 3:** Cox regression analysis of the characteristics associated with the survival of BCa patients.

Clinical characteristics	Univariate Cox regression analysis		Multivariate Cox regression analysis	
	HR (95% CI)	P Value	HR (95% CI)	P Value
**Overall survival**				
miR-96-5p (low vs. high level)	2.715 (1.342–4.587)	0.009	2.348 (1.175–3.924)	0.013
Age (≥50 vs. <50)	0.783 (0.597–1.214)	0.872	—	—
Menopause (yes vs. no)	0.695 (0.216–1.587)	0.644	—	—
Pathological Grade (III vs. I and II)	1.702 (0.348–2.475)	0.853	—	—
Her-2 (positive vs. negative)	1.405 (0.735–1.874)	0.463	—	—
PR (positive vs. negative)	1.824 (1.211–2.347)	0.031	1.041 (0.378–1.546)	0.326
ER (positive vs. negative)	0.385(0.132–1.645)	0.435	—	—
TNBC (positive vs. negative)	0.869 (0.312–1.546)	0.547	—	—
Distant metastasis (Yes vs. No)	2.577 (1.212–3.501)	0.017	1.685 (1.352–1.832)	0.037
TNM stage (III vs. II vs. I)	2.872 (1.235–3.519)	0.022	2.471 (1.578–2.659)	0.043
**Disease-free survival**				
miR-665 (high vs. low level)	2.271 (1.731–4.214)	0.013	1.897 (1.544–2.824)	0.037
Age (≥50 vs. <50)	0.884 (0.712–1.357)	0.561	—	—
Menopause (yes vs. no)	0.648 (0.126–1.624)	0.652	—	—
Pathological Grade (III vs. I and II)	1.375 (1.969–3.115)	0.024	1.674 (0.958–2.467)	0.128
Her-2 (positive vs. negative)	1.608 (1.328–3.857)	0.011	1.608 (1.244–2.879)	0.013
PR (positive vs. negative)	1.679 (1.584–3.361)	0.027	0.851 (0.164–1.379)	0.247
ER (positive vs. negative)	0.542 (0.431–1.547)	0.732	—	—
TNBC (positive vs. negative)	1.133 (0.674–2.117)	0.687	—	—
Distant metastasis (Yes vs. No)	2.247 (1.571–3.106)	0.047	1.735 (1.534–1.902)	0.041
TNM stage (III vs. II vs. I)	2.172 (1.879–4.397)	0.017	2.346 (1.984–3.406)	0.021

The bold values are less than 0.05, which have statistical significance.

### MiR-96-5p suppresses BC cell metastasis

**Figure 2:**
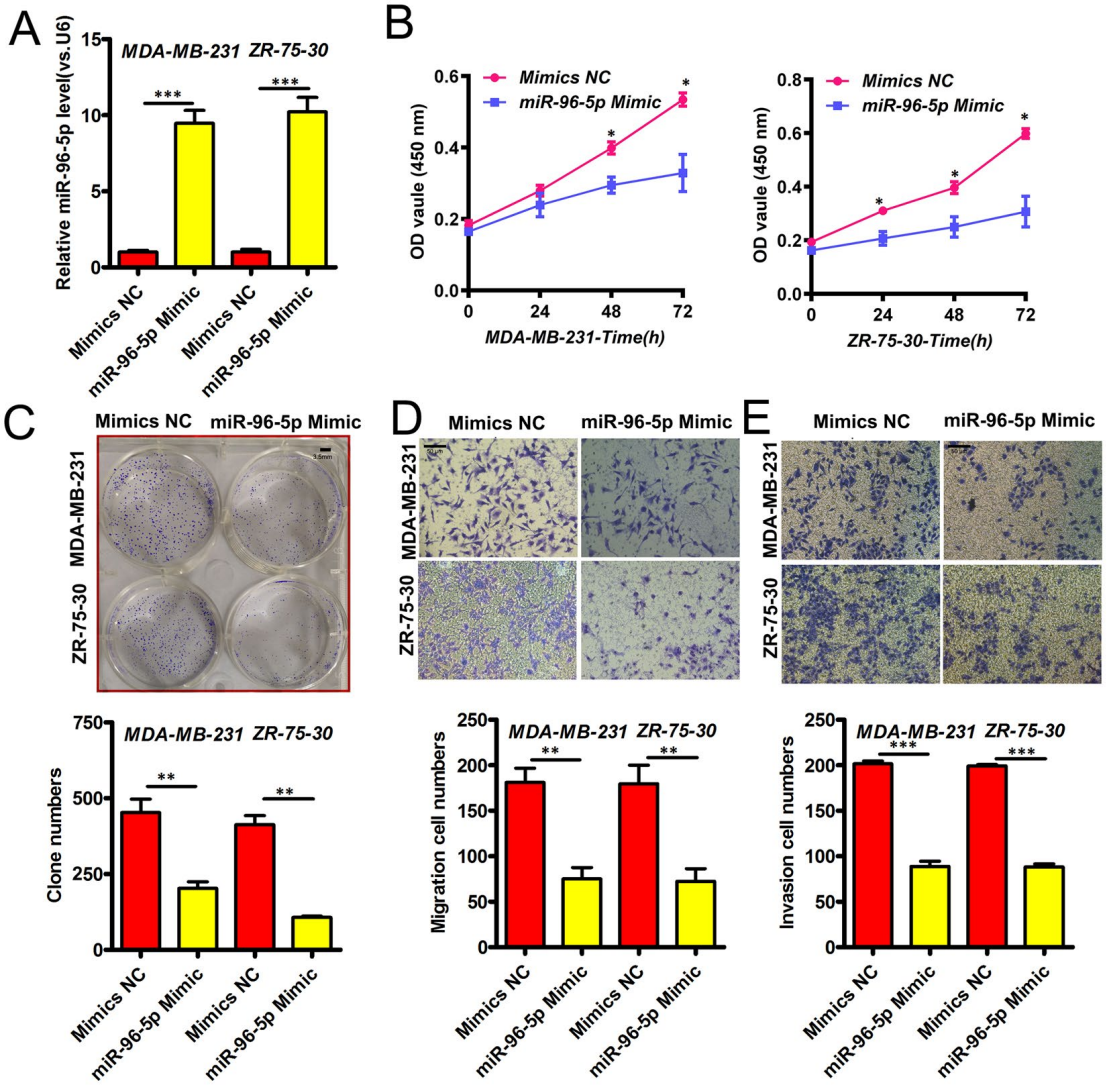
MiR-96-5p and BCa metastasis. (**A**) QRT-PCR in BCa cells expressing the indicated controls or miRNA mimics. (**B**) Cell proliferation assays following miR-96-5p overexpression. (**C**) Clonogenicity following the restoration of miR-96-5p expression. Scale bar = 3.5 mm. (**D**) Cell migration assays. Scale bar = 50 μm. (**E**) Invasiveness of BCa cells according to miR-96-5p expression. Scale bar = 50 μm. P-values as described in Fig. 1.

We next restored the levels of the miRNA through the transfection of miRNA mimics and assessed their effects on BCa cells. Successful miR-96-5p overexpression was confirmed by q-RTPCR (P < 0.05 in both cell lines, Fig. 2A) which markedly inhibited the proliferation of BCa cells (CCK-8 assays, P < 0.05 in both cell lines, Fig. 2B). Moreover, miR-96-5p overexpression inhibited the rates of colony formation (P < 0.05 in both cell lines, Fig. 2C) and reduced the migratory and invasive phenotypes of BCa cells (P < 0.05, Fig. 2D,E).

### MiR-96-5p targets CTNND1 in BCa cells

**Figure 3:**
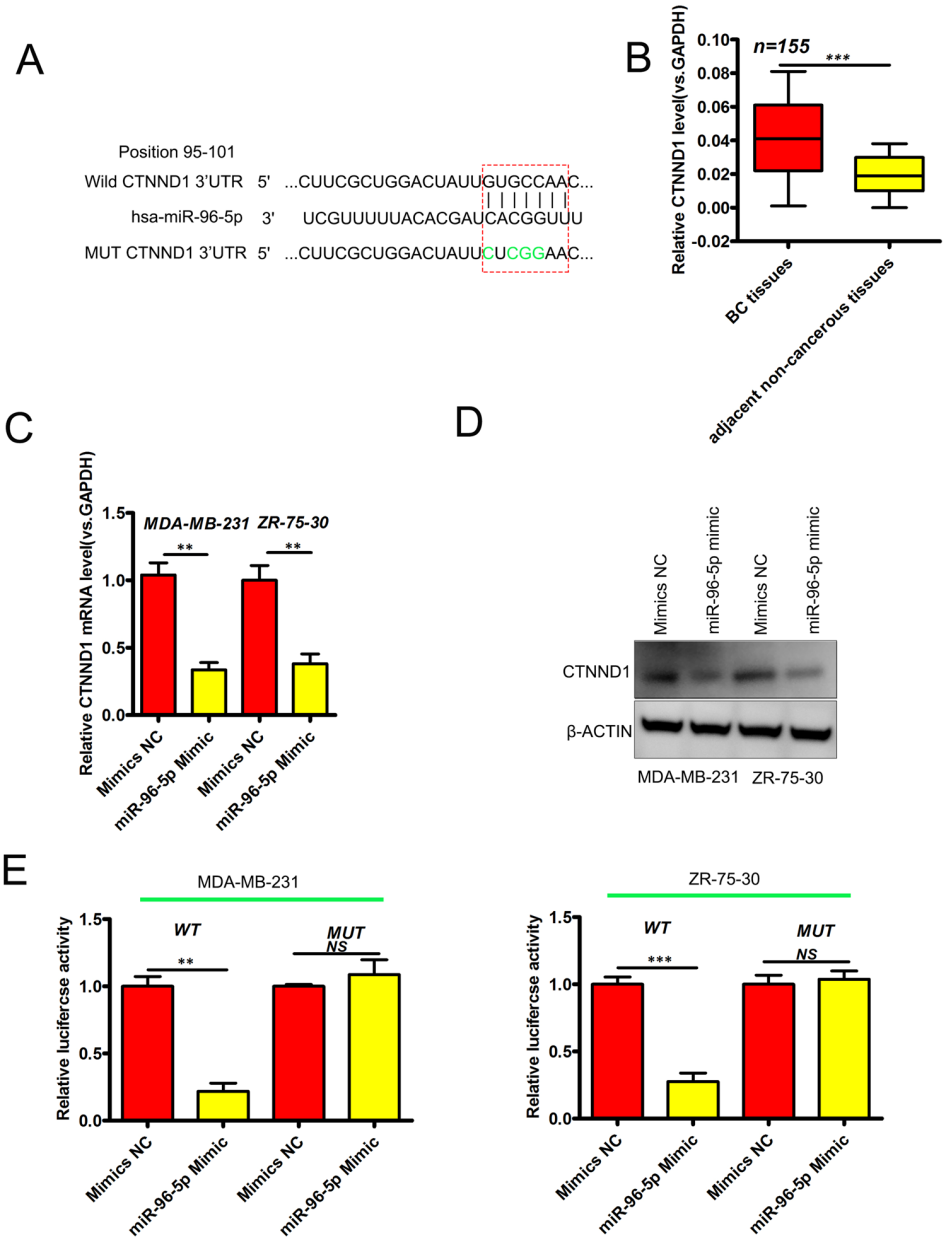
CTNND1 is a miR-96-5p target. (**A**) Binding sites in the 3′-UTR of CTNND1 mRNA. Mutated sites are underlined. (**B**) Upregulation of CTNND1 expression in BCa via qRT-PCR. *P < 0.05. (**C**,**D**) miR-96-5p upregulation decreases CTNND1 in BCa cells. N = 3, *P < 0.05. (**E**) Loss of miR-96-5p in BCa cells negatively regulates wt 3′-UTR of CTNND1 but not mt 3′-UTR. N = 3; p-values as described in Fig. 1.

Using TargetScan (http://www.targetscan.org/vert_72/) we screened a range of endogenous genes for potential miR-96-5p binding. The performed query identified that the well-characterized and important BC oncogene CTNND1 contains a complementary sequence for miR-96-5p (Fig. 3A). In view of this finding, the levels of CTNND1 were assessed in human BCa samples and adjacent non-tumor tissues. As shown in Fig. 3B, CTNND1 mRNA levels were markedly higher in BC than in normal tissue (P < 0.05). Restoring the miRNA in BCa cell-lines downregulated CTNND1 (P < 0.05; Fig. 3C,D). Luciferase reporter assays confirmed that wt CTNND1 3′-UTR cells showed reduced luciferase levels upon the restoration of miR-96-5p (P < 0.05, Fig. 3E). In control experiments, modifying the levels of miR-96-5p had no effects on luciferase activity in mt CTNND1 3′-UTR cells (Fig. 3E). This confirmed CTNND1 as the target of miR-96-5p in BCa cells.

### MiR-96-5p inhibits Wnt/β-catenin signaling through its interaction with CTNND1

**Figure 4:**
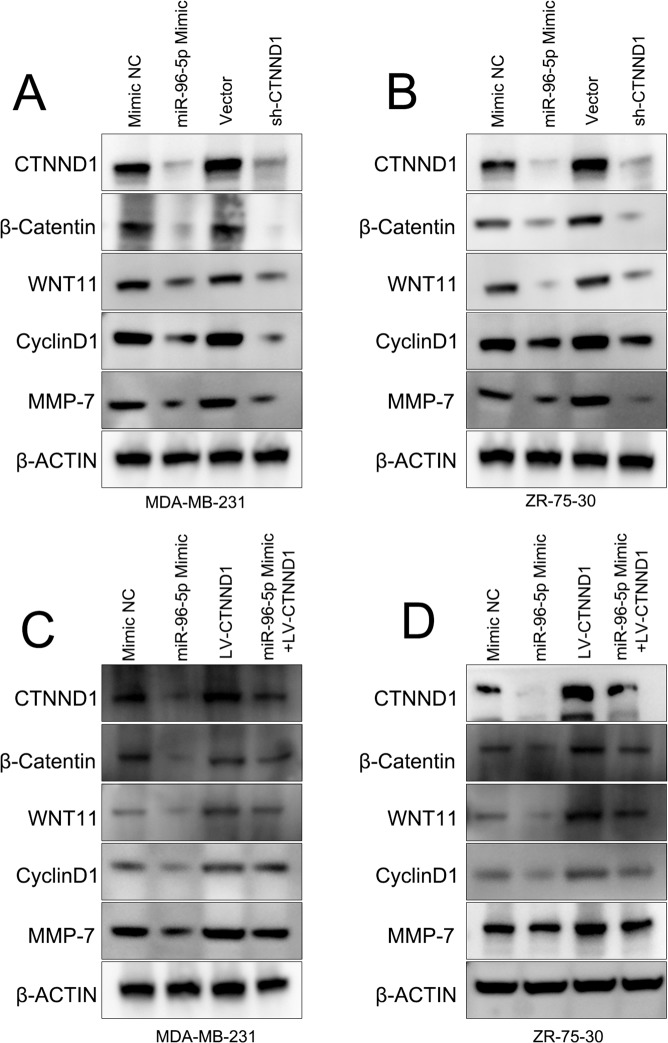
miR-96-5p regulates Wnt/β-catenin signaling through its ability to target CTNND1. (**A**,**B**) Restocration of miR-96-5p or CTNND1 silencing in the indicated BCa cell lines and their effects on the indicated signaling molecules. (**C**,**D**) MiR-96-5p inhibits Wnt/β-catenin signaling and reduces cyclin D1 and MMP7; CTNND1 restoration alleviates these effects. N = 3; P-values as described in Fig. 1.

**Figure 5:**
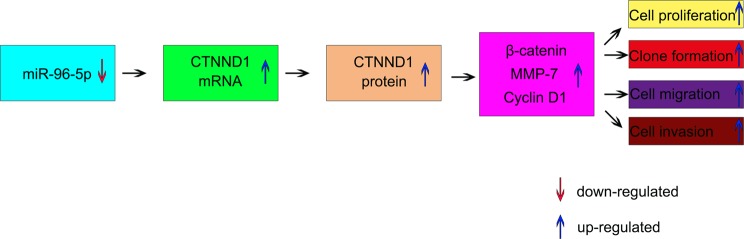
An overview of the proposed mechanism of miR-96-5p-regulated CTNND1/WNT/β-catenin signaling as a regulator of the proliferation, clone formation, migration and invasion of BCa.

MMP-7, β-catenin and CyclinD1 expression are enhanced in BCa cells and promote BCa aggressiveness^13–16^. Since CTNND1 regulates Wnt/β-catenin signaling^17^ we reasoned that this pathway is regulated by miR-96-5p in BCa cells. We therefore recovered miR-96-5p expression and performed CTNND1 silencing, and assessed the effects of these interventions on components of this pathway in BCa cells. Both resulted in a marked decrease in Wnt-β-catenin signaling (Fig. 4A,B). These results were complemented by the finding that the ectopic expression of CTNND1 expression activated Wnt/β-catenin signaling in BCa cells overexpressing miR-96-5p (Fig. 4C,D). Together, these data demonstrate unequivocally that miR-96-5p suppresses the tumor-related properties of BCa cells at least in part through CTNND1-binding and subsequent Wnt/β-catenin signaling inhibition (Fig. 5).

## Discussion

Previous study have confirmed that miRNAs play key role in the progression of BCa^18^. The multiple effects exerted by miRNAs in BCa cells include the regulation of proliferative, apoptotic, autophagic, migratory, invasive, metastatic, epithelial-mesenchymal transition (EMT), angiogenic and drug resistant phenotypes^19^. Dysregulated miR-96-5p expression occurs in numerous cancers. Here, we reveal that the downregulation of this miRNA in BCa cells and tissues that is directly associated with low RFS and OS. Low levels of the miR-96-5p were further shown to correlate with TNM and stage and importantly, distant metastasis. This highlights its anti-BCa properties and utility as a biomarker for the prediction of both survival and metastasis in BCa.

Additionally, we show that the restoration of miR-96-5p levels inhibited BCa metastatic phenotypes, highlighting its tumor suppressor function in BCa. MiRNAs bind to the 3′-UTRs of target mRNAs to reduce post-transcriptional gene expression^20^. Previous studies using various cell types have documented several targets of miR-96-5p including Caveolae1^21^, CCDC67^22^, PTEN^23^, and caspase-9^24^. The current study expanded the repertoire of its targets through the identification of CTNND1. This conclusion was supported by several experiments. In BCa tissue, CTNND1 mRNA was inversely related to miR-96-5p. Earlier reports have shown that CTNND1 is regulated by various species of miRNAs. For example, miR-145 and miR-29c target CTNND1 and prevent metastatic phenotypes in gastric cancer cells^25,26^. Direct regulation of CTNND1 by miR-409c has also been documented in osteosarcoma^27^. In BC, high expression of CTNND1 is required for tumor growth and metastasis^17^. The function of CTNND1 as an oncoprotein is dependent on its ability to indirectly activate the Wnt/β-catenin pathway, known to induce BCa progression^17,28^. In this regard, we documented that both CTNND1 silencing and miR-96-5p overexpression inhibited the Wnt/β-catenin cascade and inhibit its downstream targets such as cyclin D1 and MMP7. Of these, Cyclin D1 is required for the promotion of BC progression by Wnt/β-catenin signaling^29^, and MMP7 activity enables migration and invasion of BC cells^30^. Consistent with this notion, exogenous CTNND1 expression reversed the miR-96-5p-induced inactivation of Wnt and β-catenin, promoting metastatic phenotypes in miR-96-5p-overexpressing BCa cell lines. Together, these data highlight the critical importance of the miR-96-5p/CTNND1 axis in BCa cells. Some study limitations should be noted: (1) The study lacked *in vivo* data for further confirmation of the role of miR-96-5p on BCa; (2) We did not explore whether miR-96-5p silencing could increase MCF-10A cell migration, invasion, and proliferation through CCTNND1/Wnt-β-catenin signaling; (3) We did not explore whether miR-96-5p inhibitors produced the opposite effects to miRNA mimics in the BCa cell lines. These factors now warrant further investigation.

In summary, we demonstrate that BCa is characterized by the downregulation of miR-96-5p, and that this alteration contributes to tumor progression. MiR-96-5p post-transcriptionally suppresses CTNND1 expression thus inhibiting the metastasis of BCa cells *in vitro*. Mechanistically, miR-96-5p prevents cancer metastasis by targeting CTNND1-mediated Wnt/β-catenin signaling in BCa. The role of this miRNA for BCa therapeutics now warrants further investigation.
